# Glutamine Supplementation Enhances the Effects of a Low FODMAP Diet in Irritable Bowel Syndrome Management

**DOI:** 10.3389/fnut.2021.746703

**Published:** 2021-12-16

**Authors:** Samira Rastgoo, Nasser Ebrahimi-Daryani, Shahram Agah, Sara Karimi, Mohammad Taher, Bahram Rashidkhani, Ehsan Hejazi, Fatemeh Mohseni, Mina Ahmadzadeh, Amir Sadeghi, Azita Hekmatdoost

**Affiliations:** ^1^Department of Clinical Nutrition and Dietetics, Faculty of Nutrition and Food Technology, National Nutrition and Food Technology, Research Institute, Shahid Beheshti University of Medical Sciences, Tehran, Iran; ^2^Department of Gastroenterology and Hepatology, Tehran University of Medical Sciences, Tehran, Iran; ^3^Colorectal Research Center, Iran University of Medical Sciences, Tehran, Iran; ^4^Gastroenterology and Liver Diseases Research Center, Research Institute for Gastroenterology and Liver Diseases, Shahid Beheshti University of Medical Sciences, Tehran, Iran

**Keywords:** irritable bowel syndrome (IBS), glutamine, diet, low FODMAP diet, clinical trial

## Abstract

**Background and Aims:** Although irritable bowel syndrome is one of the most common gastrointestinal disorders presented to gastroenterologists, therapeutic strategies are not yet well-established. Accordingly, we conducted a randomized, double-blind, placebo-controlled, clinical trial to evaluate the possible superiority of adding glutamine supplement to low fermentable oligo- di- monosaccharides and polyols (FODMAP) diet in patients with irritable bowel syndrome (IBS).

**Methods:** Eligible adults were randomized to receive a low FODMAP diet either with glutamine (15 g/day) or a placebo for 6 weeks. The primary endpoint was a significant reduction in IBS-symptom severity score (IBS-SSS). Secondary endpoints were changes in IBS symptoms, stool frequency, consistency, and quality of life.

**Results:** The study group enrolled 50 patients, among which 22 participants from each group completed the study protocol. The glutamine group had significant changes in total IBS-severity score, dissatisfaction of bowel habit and interference with community function (58% reduction; *P* < 0.001, 57% reduction; *P* < 0.001, 51% reduction; *P* = 0.043, respectively). Improvement in IBS-severity score of more than 45% was observed in 22 of 25 participants (88%) in the glutamine group, while it was only 15 of 25 participants (60%) in the control group (*p* = 0.015). No serious adverse events were observed.

**Conclusions:** Our findings indicated the superiority of adding glutamine supplementation to a low FODMAP diet in amelioration of IBS symptoms while confirming the beneficial effects of a low FODMAP diet in IBS management.

## Introduction

Although irritable bowel syndrome is one of the most prevalent referrals to gastroenterologists, the best method for managing it is still unknown (1, 2). The high prevalence of irritable bowel syndrome (IBS) besides suboptimal medical treatments leads to significant economic costs and psychosocial burden (3–5). The pathophysiology of IBS is multifactorial and the molecular mechanisms underlying the pathophysiology of IBS are not well-understood. However, dietary intolerance, alternation in gut microbiota, and increased intestinal permeability have been suggested as potential risk factors (3, 6, 7). Recent studies have shown that a diet low in fermentable oligosaccharides, disaccharides, monosaccharides, and polyols (FODMAPs) can improve gastrointestinal symptoms in patients with IBS (8–10); however, the patients on low FODMAPs diet are not completely free of symptoms (11). Moreover, the results of previous studies have mainly reported the efficacy of a low FODMAP diet on pain and bloating reduction, with no effect on stool consistency and frequency (8). Thus, it seems that we still need new strategies for IBS management with a special focus on stool consistency and frequency.

Glutamine is a non-essential amino acid that is a preferred energy source for cells with rapid turnover such as lymphocytes and enterocytes. This amino acid promotes enterocyte proliferation, regulates tight junction proteins, and suppresses pro-inflammatory signaling pathways (12). It has been reported that the increased intestinal permeability that occurs in diarrhea-predominant patients with IBS might be due to decreased glutamine synthetase levels (13). Meanwhile; experimental evidence suggests that glutamine supplementation reduces intestinal permeability (14, 15). Furthermore, glutamine supplementation changes the intestinal microenvironment and regulates intestinal bacteria's utilization and metabolism of amino acids, thereby altering the composition of intestinal microbiota (16, 17). Modulation of intestinal microbiota might ameliorate constipation and improve intestinal function (18, 19).

In light of this experimental evidence, we hypothesized that the co-administration of a low FODMAP diet and an oral glutamine supplement would reduce the symptoms and improve the quality of life of patients with IBS more effectively than a low FODMAP diet alone. Thus, the current randomized, double-blind, placebo-controlled trial was conducted to evaluate the effect of a low FODMAP diet with glutamine supplementation on patients with IBS' clinical outcomes and quality of life.

## Methods and Materials

### Participants

This study's participants comprised patients with IBS (as defined by Rome IV criteria) (20) without any other disorders aged between 18 and 70 years old with body mass index (BMI) ranging from 18.5 to 25. Patients were recruited between June 2020 and December 2020 from two gastroenterology clinics in Tehran, Iran. Patients who did not have the following disorders were eligible to participate in the study: any organic intestinal diseases based on colonoscopy over the past 5 years, intestinal infection, history of colorectal disorders, major intestinal surgery, liver, kidney, psychiatric disease, or any GI disease other than IBS.

Patients were excluded from the study if they (a) were taking any medication with antispasmodics, antibiotics, anti-diarrhea or laxative properties, prokinetics, non-steroidal anti-inflammatory, and immunosuppressive agents or (b) were a smoker, pregnant, or breastfeeding. Patients with known allergies to glutamine or whey protein were excluded, as were those who were taking or had taken supplements containing glutamine or whey protein. Patients were also excluded if they had consumed synthetic sweeteners within 2 days before the study or during the study, as such sweeteners can alter intestinal permeability. Finally, any patients unwilling to adhere to the recommended diet were also excluded.

Patients were sub-classified as either having predominant diarrhea (IBS-D), predominant constipation (IBS-C), mixed or alternating bowel habits (IBS-M), or undetermined categories (IBS-U). All patients provided informed written consent to participate in the study after the study protocol was thoroughly explained.

### Study Design and Intervention

This study was a randomized, double-blind, placebo-controlled trial. Fifty patients who met the inclusion criteria were randomly assigned to either the experimental (glutamine) or control (placebo) group. Randomization was based on a random table sequence, and all investigators were blind regarding which patients were in which group.

Since receiving 15 g of glutamine or whey does not cause side effects for patients (21), participants received an oral glutamine powder or placebo powder (whey protein) at a dose of 15 g (5 g mixed in water three times per day) for 6 weeks. The powders were similar in color, consistency, and taste. The supplements were concealed as A or B by a third party—for the duration of the study, neither the participants nor investigators knew which group was taking which supplement.

All participants were advised to follow a low FODMAP diet in addition to taking the assigned supplement. Diets were administered by an experienced dietitian according to the guidelines of the National Institute for Health and Care Excellence (NICE) while omitting high FODMAPs foods. All diets contained <5-g FODMAPs per day. Patients' adherence to the diet was evaluated by recording of 3 days (1 weekend and 2 workdays) dietary recalls at week 2 and the end of the study.

Dietary intakes of FODMAPs were assessed using Monash University's low-FODMAP diet database and quantitative reports of FODMAP content in recent studies (22, 23). Patients were followed by phone calls to assess protocol adherence, record any supplement side effects, and answer any study-related questions. Patients' competency was assessed using the measurement of remained supplements.

### Study Outcomes

The primary outcome was a significant reduction in IBS-symptom severity score (IBS-SSS). Secondary outcomes were changes in IBS symptoms, quality of life, and stool consistency and frequency. All clinical outcomes were evaluated at baseline and the end of the study using the IBS-SSSQ (24). This instrument includes five clinically relevant items that determine the severity of abdominal pain, the frequency of pain, abdominal distension, satisfaction with bowel habits, and the interference of IBS with community function as measured on the visual analog scale (VAS) (with an array of 100 mm, with 0 indicating no symptoms and 100 representing extremely severe symptom). The sum of these five items was IBS- symptom severity score (range = 0–500). Scores of 75–175, 175–300, and > 300 indicated mild, moderate, and severe cases, respectively.

Each patient's quality of life (QoL) was assessed at baseline and the end of the study using a 34-item self-report measure specific to IBS (IBS-QoL) (25). Each item was answered on a 5-point Likert scale, and participants' scores were summed to derive the overall score. Scores were subsequently transposed onto a scale from 0 to 100, with higher scores indicating a better quality of life.

Stool consistency was assessed using the validated Bristol Stool Form Scale (BSFS) (26). Stool frequency (i.e., number of stools per day) was recorded at baseline and the end of the study.

### Statistical Analysis

The sample size was calculated based on the formula by considering α (type 1 error) = 0.05 and power of 80% according to the published article (11), which was obtained 21. Therefore, according to the formula 21 subjects were needed in each group, considering the probable dropouts, we assigned 25 subjects for each group to meet the adequate power.

All hypothesis tests were 2-tailed, with *P* < 0.05 denoting statistical significance. The Kolmogorov–Smirnov-test with a significance level of 5% was used to test continuous variables for the normality assumption. The chi-square test or Fisher exact-test was used to determine the differences of categorical variables between groups. Comparison between the variables was performed by paired-samples *t*-test/Wilcoxon at the beginning and end of the study in each group. To detect differences in continuous variables between the two groups, independent-samples *t*-test/Mann-Whitney was used. The main effects and interaction effects of the interventions were compared between groups using analysis of covariance (ANCOVA) with baseline measures as a covariate. The data were analyzed according to the intention-to-treat principle. Patients missing the final data were imputed. A multiple imputation procedure was used based on multiple imputations by chained equation. In the multiple imputation procedure, five imputed data sets were generated. The results of the five imputed data sets were pooled to obtain data estimates.

Collected information by food diaries was analyzed in the Nutritionist 4 software modified for Iranian foods (Karen Pharma & Food Supplement Co., Tehran, Iran). Data from the food-record questionnaires were entered and analyzed by an expert dietitian.

### Ethics and Approvals

The study protocol was approved by Shahid Beheshti Ethics Committee (IR.SBMU.NNFTRI.REC.1398.083), and it was registered at the Iranian Registry of Clinical Trials, with the registration number IRCT20100524004010N28.

## Results

### Patient Characteristics

**Figure 1:**
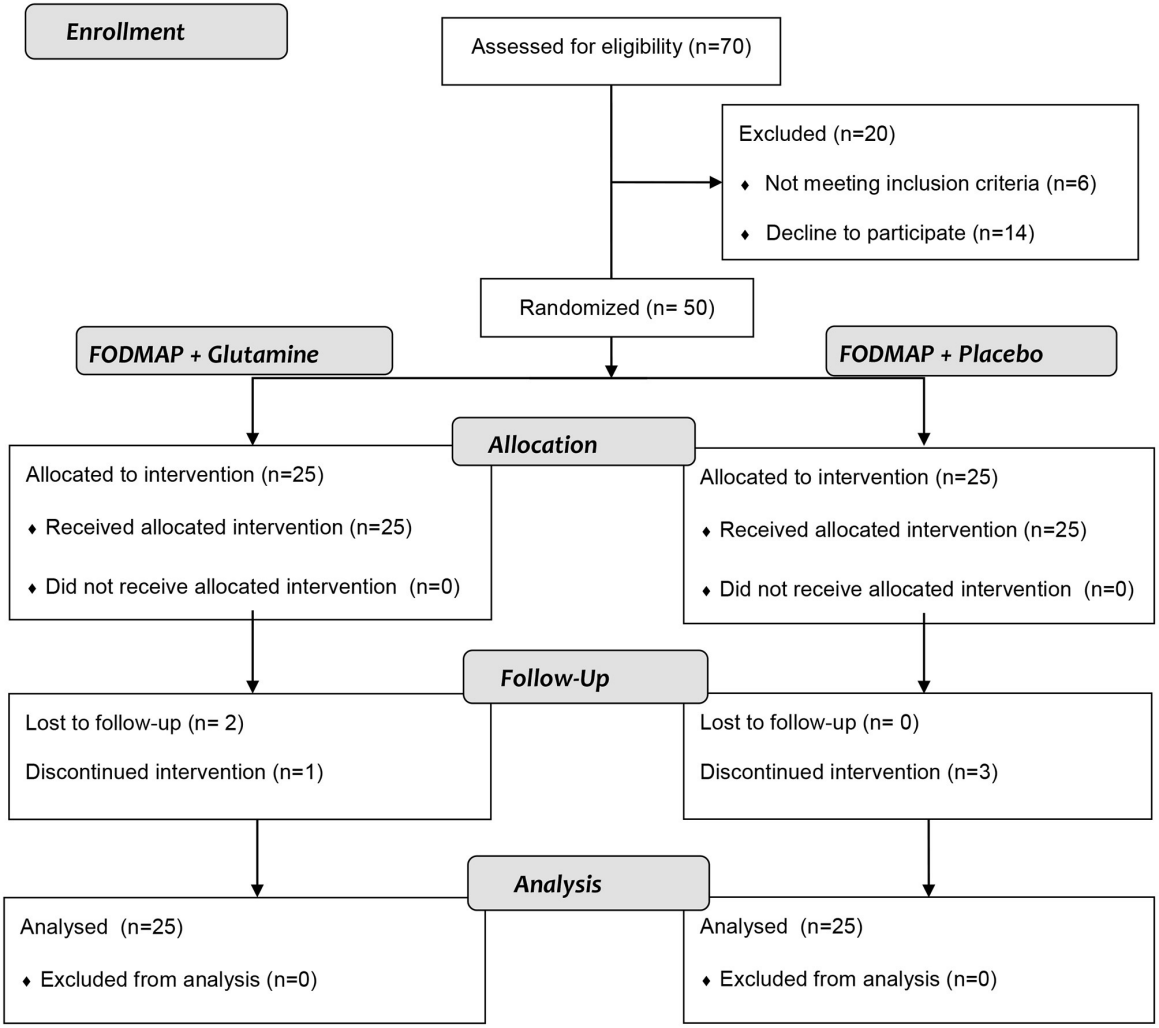
Consort flow chart of the study.

Between June 2020 and December 2020, 70 patients were recruited from two gastroenterology clinics and screened for this trial. From this initial group, 20 patients were excluded and the other 50 were enrolled and underwent the randomization process. Among the 50 included patients, 25 were assigned to the glutamine group and 25 were assigned to the placebo group. One patient was excluded from the glutamine group after discontinuing the study protocol, and two were lost to follow-up issues. Meanwhile, three patients from the control group were excluded due to non-compliance with the recommended diet. Therefore, 22 patients in the glutamine group and 22 patients in the control group completed the study protocol (Figure 1).

Table 1 shows the baseline characteristics of participants. The groups were similar in all characteristics except abdominal pain frequency (*p* = 0.022).

**Table 1:** Characteristics of participants with irritable bowel syndrome at the baseline.

**Baseline characteristics**	**Glutamine (*n* = 25)**	**Control (*n* = 25)**	***P*-value**
Age (years)^a^	40.36 ± 15.15	35.09 ± 8.53	0.256
Sex—no. (%)^b^			0.540
Female	14 (56%)	16 (64%)	
Male	11 (44%)	9 (36%)	
Weight (kg)^a^	67.36 ± 8.44	63.09 ± 9.61	0.125
BMI (kg/m^2^)^a^	23.48 ± 1.57	22.71 ± 1.62	0.118
IBS subtype—no. (%)^b^			0.215
Constipate	3 (12%)	4 (16%)	
Diarrhea	16 (64%)	14 (56%)	
mixed	6 (24%)	4 (16%)	
Unclassified	0	3 (12%)	
IBS symptoms^a^			
IBS-SSS	308.41 ± 82.44	278.86 ± 47.71	0.155
Abdominal pain intensity	65.91 ± 25.80	56.36 ± 15.44	0.092^*^
Abdominal pain frequency	52.73 ± 29.14	35 ± 18.71	0.022^*^
Abdominal distension	56.14 ± 22.30	66.82 ± 12.20	0.292^*^
dissatisfaction with bowel habits	64.32 ± 29.93	49.77 ± 20.73	0.068
Interference with life	69.32 ± 22.43	70.91 ± 10.19	0.971^*^
Stool frequency (no./day)	2.95 ± 2.19	2.68 ± 2.05	0.685^*^
Stool consistency	5.14 ± 1.70	4.55 ± 1.62	0.240^*^
Quality of life^a^	63 ± 19.99	65 ± 10.44	0.652

a Data are reported as mean ± SD and compared by independent sample t-test/Mann-Whitney test (^*^).

b *Data are reported by n (percentage of total); the chi-square test was used*.

*Statistically significant values in bold*.

### Nutritional Data

There was no significant difference in the nutritional compositions of the diet between the two groups (Table 2). All participants' dietary recalls demonstrated acceptable adherence to the diet.

**Table 2:** The mean daily nutrition information during the study in intervention groups.

**Parameter**	**Glutamine group (*****n*** **=** **25)**		**Control group (*****n*** **=** **25)**			
	**Week 2**	**Week 6**	**Week 2**	**Week 6**	***P* ^1^**	***P* ^2^**
Energy (kcal)	1,799.64 ± 231.60	1,772.05 ± 226.64	1,888.5 ± 302.57	1,885.95 ± 285.05	0.280	0.286
Protein (gr)	73.53 ± 18.86	71.81 ± 22.96	81.30 ± 17.99	77.96 ± 16.64	0.170	0.315
Fat (gr)	67.40 ± 12.99	67.14 ± 14.15	67.08 ± 13.59	65.45 ± 12.29	0.936	0.778
Carbohydrates (gr)	224.61 ± 39.10	223.33 ± 39.33	242.26 ± 35.53	239.70 ± 44.88	0.139	0.205
Dietary fiber (gr)	11.32 ± 3.18	11.12 ± 3.02	12 ± 2.86	11.71 ± 3.64	0.461	0.557
Lactose (gr)	0.822 ± 1	0.622 ± 1.02	0.479 ± 0.69	0.277 ± 0.65	0.285	0.222
Excess fructose (gr)	0.150 ± 0.13	0.143 ± 0.09	0.272 ± 0.26	0.168 ± 0.13	0.051	0.953
Polyols (gr)	0.239 ± 0.33	0.191 ± 0.34	0.327 ± 0.35	0.254 ± 0.41	0.245	0.662
GOS (gr)	0.125 ± 0.09	0.150 ± 0.08	0.131 ± 0.05	0.161 ± 0.08	0.798	0.657
FOS (gr)	0.131 ± 0.08	0.186 ± 0.10	0.138 ± 0.06	0.193 ± 0.08	0.743	0.813
Total fructans (g)	0.475 ± 0.32	0.599 ± 0.28	0.507 ± 0.18	0.644 ± 0.30	0.418	0.613
Total FODMAP(g)	1.81 ± 1.36	1.70 ± 1.15	1.72 ± 0.87	1.50 ± 1.03	0.805	0.489

*Data are reported as mean ± SD and compared by sample t-test*.

*P^1^: between group at week 2*.

*P^2^: between group at week 6*.

*GOS, galaco-oligosaccharide; FOS, fructo-ologosaccharides; FODMAP, fermentable oligo- di- mono- saccharides and polyols. Statistically significant values are bolded*.

### Symptoms

**Figure 2:**
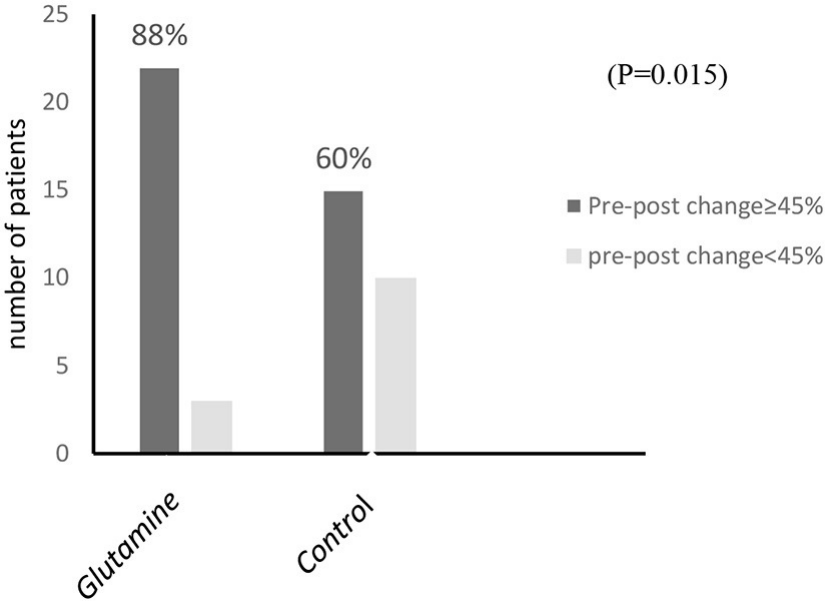
The number of patients in both glutamine and control groups at the end of the study based on the percentage of change in IBS severity score.

Improvement in IBS-severity score of more than 45% was observed in 22 of 25 participants (88%) in the glutamine group, while it was only 15 of 25 participants (60%) in the control group (*P* = 0.015; Figure 2). The total scores of IBS-SS and the scores for individual items (abdominal pain intensity, abdominal pain frequency, abdominal distension, dissatisfaction with bowel habits, and interference with life), stool frequency, and consistency are shown in Table 3. Significant improvements were observed in these variables in both groups at the end of the study compared with the baseline (*p* < 0.001 for total scores and individual items of IBS-SSS and *P* = 0.002, *P* = 0.003, respectively, for stool frequency and consistency).

**Table 3:** Gastrointestinal symptoms and quality of life in patients with irritable bowel syndrome.

**Parameter**	**Glutamine group (*****n*** **=** **25)**			**Control group (*****n*** **=** **25)**		
	**Baseline**	**Week 6**	***P* ^1^**	**Baseline**	**Week 6**	***P* ^2^**
Total scores of IBS-SSS	308.41 ± 82.44	128.41 ± 41.30	<0.001	278.86 ± 47.71	151.59 ± 32.12	<0.001
Abdominal pain intensity	65.91 ± 25.80	29.09 ± 14.36	<0.001	56.36 ± 15.44	29.55 ± 11.64	<0.001
Abdominal pain frequency	52.73 ± 29.14	20 ± 10.69	<0.001	35 ± 18.71	18.64 ± 8.33	<0.001
Abdominal distension	56.14 ± 22.30	19.55 ± 8.58	<0.001	66.82 ± 12.20	26.14 ± 9.50	<0.001
dissatisfaction of bowel habits	64.32 ± 29.93	26.14 ± 14.63	<0.001	49.77 ± 20.73	35.68 ± 16.49	<0.001
Interference with life	69.32 ± 22.43	33.64 ± 13.64	<0.001	70.91 ± 10.19	41.59 ± 9.31	<0.001
Stool frequency (no./day)	2.95 ± 2.19	1.59 ± 0.796	0.002	2.68 ± 2.06	1.68 ± 0.894	0.002
Stool consistency	5.14 ± 1.70	4.00 ± 0.617	0.003	4.55 ± 1.62	3.82 ± 0.907	0.003
Quality of life	63.23 ± 19.99	76.32 ± 16.74	<0.001	65.41 ± 10.44	76.45 ± 9.86	<0.001

*Values are reported as mean ± SD*.

*P^1^: Obtained from the Wilcoxon test for comparison of data between the beginning and end of the study*.

*P^2^: Obtained from the paired samples t-test for comparison of data between the beginning and end of the study*.

Table 4 shows the comparison of pre-post-treatment changes between the glutamine and placebo groups. The glutamine group had significant changes in total IBS-severity score, dissatisfaction of bowel habit and interference with community function (58% reduction; *P* < 0.001, 57% reduction; *P* < 0.001, 51% reduction; *P* = 0.043, respectively). No adverse effect was reported in any group of interventions.

**Table 4:** Effects of intervention on studied outcomes.

**Parameter**	**Pre-post change (%)**		***P*^1^-value**	***P*^2^-value**
	**Glutamine (*n* = 25)**	**Control (*n* = 25)**		
Total scores of IBS-SSS	58% (–)	46% (–)	**<0.001**	**<0.001**
Abdominal pain intensity	56% (–)	48% (–)	0.072	0.195
Abdominal pain frequency	55% (–)	41% (–)	**0.027**	0.074
Abdominal distension	61% (–)	61% (–)	0.522	0.742
Dissatisfaction of bowel habits	57% (–)	28% (–)	**<0.001**	**<0.001**
Interference with life	51% (–)	41% (–)	**0.002**	**0.043**
Quality of life	27% (+)	18% (+)	0.205	0.327
Stool frequency (no./day)	39% (–)	29% (–)	0.182	0.194
Stool consistency	9% (–)	8% (–)	0.152	0.227

*P^1^-value: Obtained from two-sample t-test*.

*P^2^-value: Obtained from ANCOVA test adjusted for the baseline values between studied groups. Statistically significant values are bolded*.

### Quality of Life

Baseline quality of life scores was not significantly different between the two groups (Table 1). The QOL scores increased in both intervention groups (+13 ± 8.12, *P* < 0.001 in the glutamine group and +11 ± 5.12, *P* < 0.001 in the control group) at the end of the study compared with the baseline (Table 3). No significant difference was observed between groups at the end of the study (*P* = 0.353).

## Discussion

The present study demonstrated the superiority of adding a glutamine supplement to a low FODMAP diet in amelioration of IBS symptoms while also confirming the beneficial effects of low FODMAPs diets in IBS management (9).

It is well-known that a low FODMAPs diet ameliorates IBS symptoms by reducing luminal distension owing to the osmotic effects of FODMAPs and their rapid fermentation preferentially to hydrogen (1, 10, 27). However, several studies have reported that increased intestinal permeability is another mechanism underlying IBS symptoms (7, 28). Moreover, it has been shown that this disturbance in gut integrity is related to decreased glutamine synthetase levels in patients with IBS (13). This, in turn, results in visceral hypersensitivity, leading to increased gastrointestinal symptoms (7, 13). Experimental evidence has also shown that glutamine improves intestinal permeability (29, 30).

On the other hand, low-grade inflammation has been observed in the intestinal mucosa of patients with IBS, especially those with increased intestinal permeability (31). Evidence shows that glutamine has anti-inflammatory properties, as it inhibits the activation of nuclear factor κB (NF-κB), signal transducer and activator of transcription (STAT), and inflammatory cytokines such as IL-6, TNF-α, and IL-8 (12).

Zhou et al. (32) reported that the up-regulation of microRNA29 in the colonic mucosa of patients with IBS-D reduces claudin-1 levels, which leads to increased intestinal permeability. Claudin-1 is an integral component of the structure of tight junctions and plays a crucial role in regulating epithelial barrier function (33). Therefore, the alteration of tight junction proteins might initiate IBS and contribute to visceral hypersensitivity (34, 35). Meanwhile, in an *ex-vivo* study evaluating the effects of glutamine on claudin-1 tight junction proteins, colonic biopsies from patients with IBS-D were incubated in cell cultures with glutamine, and the results indicated increased claudin-1 expression (14).

Studies investigating the effects of glutamine supplementation on the severity of gastrointestinal symptoms in patients with IBS are scarce. In line with our study, Zhou et al. (21) found that oral glutamine supplementation normalized intestinal permeability and improved gastrointestinal symptoms in post-infectious IBS. In this study, the patients did not receive dietary advice, so the changes in IBS symptoms were less than our study.

Moreover, recent studies have reported the potential role of intestinal microbiota in the pathophysiology of IBS (36–38). For instance, gut microbiota can affect motor function, hypersensitivity, and immune activity in the gut (resulting in low-grade inflammation), leading to the development of IBS or the exacerbation of gastrointestinal symptoms (37, 39). A recent review of *clinical, in vitro*, and *in vivo* studies reported that glutamine affects gut microbiota community and composition through several mechanisms. Therefore, it can be used to manage some conditions such as bacterial translocation, inflammation, and constipation (18). Furthermore, Zhang et al. reported that glutamine supplementation in constipated animals improves intestinal function and ameliorates constipation by modulating gut microbiota through increasing intestinal friendly microbiota levels (19).

The protective and therapeutic roles of glutamine have also been reported in clinical trials for other gastrointestinal diseases (40–42). Glutamine could possibly improve the condition of patients with IBS by regulating intestinal permeability *via* increased tight junction proteins expression, modulating the inflammatory response, oxidative stress, or innate immune response, and also altering intestinal microbiota (29).

This study has some advantages; According to our research, this is the first clinical trial that assessed the superiority of adding glutamine supplement to low FODMAPs in the management of patients with IBS. Preparing individually low FODMAPs diet, assessment of dietary composition, FODMAPs content, and also assessment of dietary adherence during the intervention were other strengths of this study. A limitation of this study was that patients were not followed up after the study ended. Given that national local factors affect the composition of FODMAPs in foods (27), measuring the content of FODMAPs using non-localized data was another limitation of our study.

## Conclusion

In conclusion, this randomized, double-blind, placebo-controlled trial has shown the superiority of adding glutamine supplementation to a low FODMAPs diet in amelioration of IBS symptoms, while confirming the beneficial effects of a low FODMAPs diet in IBS management. Further studies are needed to find the optimum dosage of glutamine supplementation for IBS management.

## Data Availability Statement

The raw data supporting the conclusions of this article will be made available by the authors, without undue reservation.

## Ethics Statement

The studies involving human participants were reviewed and approved by NNFTRI. The patients/participants provided their written informed consent to participate in this study.

## Author Contributions

SR and AH: conceptualized and designed the study and wrote the manuscript. SR and BR: analyzed the data. SR, NE-D, SA, FM, MA, MT, and SA: collected the data. EH and AH: interpreted the data and provided professional comments. AH: critically revised the manuscript for intellectual content, data accuracy, and had responsibility for the final content. All authors have read and approved the final manuscript.

## Funding

This study was financially supported by Shahid Beheshti University of Medical Sciences, Tehran, Iran. The funder did not play any role in study design and interference.

## Conflict of Interest

The authors declare that the research was conducted in the absence of any commercial or financial relationships that could be construed as a potential conflict of interest.

## Publisher's Note

All claims expressed in this article are solely those of the authors and do not necessarily represent those of their affiliated organizations, or those of the publisher, the editors and the reviewers. Any product that may be evaluated in this article, or claim that may be made by its manufacturer, is not guaranteed or endorsed by the publisher.
